# Neuroendoscopic surgery in neonates — indication and results over a 10-year practice

**DOI:** 10.1007/s00381-021-05272-y

**Published:** 2021-07-03

**Authors:** Andreas Schaumann, Christoph Bührer, Matthias Schulz, Ulrich-Wilhelm Thomale

**Affiliations:** 1 Pediatric Neurosurgery, Charité Universitätsmedizin Berlin, Augustenburger Platz 1, 13353 Berlin, Germany; 2 Department of Neonatology, Charité Universitätsmedizin Berlin, Berlin, Germany

**Keywords:** Neuroendoscopy, Neonates, Neuroendoscopic lavage, Posthemorrhagic hydrocephalus, Postinfectious hydrocephalus, Intraventricular hemorrhage, Intraventricular infection, Ventricle peritoneal shunt

## Abstract

**Purpose:**

Neuroendoscopic procedures for treatment of term and preterm newborn infants, such as endoscopic lavage for posthemorrhagic hydrocephalus, are gaining popularity despite sparse data. This single-institution report compiles all neuroendoscopic surgical procedures performed in neonates during a 10-year period.

**Methods:**

Charts and electronic records were reviewed of all consecutive newborns who underwent a neuroendoscopic procedure before reaching a postmenstrual age of 44 weeks between 09/2010 and 09/2020. Available documentation was reviewed regarding the performed neuroendoscopic procedure, course of disease, complications, and all re-operations throughout the first year of life.

**Results:**

During the 10-year study period, 116 infants (median gestational age at birth: 29 ^1^/7 weeks) underwent a total of 153 neuroendoscopic procedures (median postmenstrual age at surgery: 35 ^0^/7 weeks). The most common indication at the time of the neuroendoscopic procedures (n = 153) was intraventricular hemorrhage (IVH, n = 119), intraventricular infection (n = 15), congenital malformation (n = 8), isolated 4th ventricle (n = 7), multiloculated hydrocephalus (n = 3), and tumor (n = 1). Thirty-eight of 116 children (32.8%) underwent 43 operative revisions after 153 neuroendoscopic procedure (28.1%). Observed complications requiring surgical revision were secondary infection (n = 11), CSF fistula (n = 9), shunt dysfunction (n = 8), failure of ETV (n = 6), among others. 72 children (62%) of 116 children required permanent CSF diversion via a shunt. The respective shunt rates per diagnosis were 47 of 80 (58.8%) for previously untreated IVH, 11 of 13 (84.6%) for intraventricular infection. Shunt survival rate for the first year of life was 74% for the whole cohort.

**Conclusion:**

The experience with this large cohort of neonates demonstrates the feasibility of neuroendoscopic technique for the treatment of posthemorrhagic or postinfectious hydrocephalus. Rate and type of complications after neuroendoscopic procedures were within the expected range. Assessing the potential long-term benefits of neuroendoscopic techniques has to await results of ongoing studies.

## Introduction

The application of neuroendoscopic surgery has gained widespread utilization to treat a wide variety of intracranial and intraspinal pathologies especially in pediatric neurosurgical practice. While a multiplicity of data about neuroendoscopic interventions in children has been published, data concerning neonates remains scarce [1–4].

In general, the spectrum of performed neurosurgical interventions in this age group is limited and usually related to disturbed CSF circulation. Because of the fragility of the treated children, temporary treatment options — e.g., insertion of ventricular access device (VAD) or ventriculo-subgaleal shunts (VSGS) — are applied to bridge the time until definite surgeries can be performed. Despite the complex condition of neonates, the application of neuroendoscopic techniques has been introduced and gained some popularity for specific indications [5]. The primary application is to treat intraventricular hemorrhage and subsequent hydrocephalus [6–8]. However, no comprehensive series of possible neuroendoscopic surgical indications in this age group has been described.

Therefore, the aim of the presented manuscript is to review all neuroendoscopic surgeries performed in neonates in our institution over a 10-year period. The report on all applied indications for neuroendoscopic procedures, the differing applied surgical techniques as well as on outcome data after surgery and on experienced complications allows a balanced review of possible merits and problems in this specific age group.

## Material and methods

### Patient cohort

A retrospective search of the hospital data system was performed to identify all neonates who underwent a neuroendoscopic procedure during the 10-year study period between 09/2010 and 09/2020. Only children who underwent neuroendoscopic surgery before the completion of the neonatal period (before the 28th day of life) were included for this study. For all identified children the respective age was corrected by the documented estimated day of delivery (EDD) to assure accurate inclusion of individuals.

Patient files and operative notes of all children fitting the defined inclusion criteria were reviewed regarding the performed neuroendoscopic procedure, the experienced course of disease, the possible complications, and all re-operations throughout the first year of life.

### Clinical management

All surgical procedures were performed under general anesthesia with the head fixed in a vacuum mattress. Prophylactic antibiotics were administered prior. Generally, the access into the ventricular system was performed under transfontanellar ultrasound guidance. For lavage procedures, the Minop or Lotta endoscope (Aesculap or Storz, Germany) was used to allow sufficient irrigation; for fenestration procedures, the Little Lotta Endoscope (Storz, Germany) was used because of its smaller diameter. The surgical technique for lavage has been extensively described previously [5, 8]. Briefly, through the lateralized frontal, pre-coronal burr hole the whole anterior–posterior dimension of the lateral ventricle from the frontal to the occipital horn could be reached. Turbid intraventricular fluid was irrigated until visualization of anatomic landmarks was possible and any solid hematoma remnants were actively aspirated in a piece-meal fashion via the outflow channel using a 10 cc syringe connected by a “Heidelberger” tubing system to the enoscope's Luer lock outflow connector. Because of the rigid endoscope any hematoma remnants in the temporal horns were beyond direct reach, but may have been dislocated by irrigation and may thus be subsequently aspirated. An interventricular septostomy allowed equal access of the contralateral ventricle. After removal of the endoscope the remaining transcortical channel to the ventricle was sealed with a gelatin plug wrapped around a ventricular catheter and connected to a subcutaneous reservoir to allow possible subsequent CSF punctures. Other inserted catheters for transaqueductal stents or ventricular catheters of a ventriculo-peritoneal shunt were treated likewise.

In general, we did not indicate endoscopic ventriculocisternostomy (ETV) in this age group due to the expected extremely low success rate. Thereby additional risk exposure to the patient should be avoided. Exceptions from this rule might have been made due to the individual neurosurgeon´s decision in proven non-communicating hydrocephalus as visualized by pressure gradient at the membranes of the 3rd ventricle in order to prolong the time to shunt insertion.

The wound was meticulously closed in three layers with adaptation of the periosteum across the reservoir, subcutaneous sutures, and a running skin closure. For all shunts a programmable valve with gravitational assistance was used (proGAV or proGAV 2.0, Miethke, Germany). All catheters were antibiotically impregnated with clindamycin and rifampicin (Bactiseal, Codman-Integra, USA).

All children were postoperatively admitted to the neonatal intensive care unit and were followed with daily clinical assessments. Head circumference measurements and serial ultrasounds until discharge were regularly done. If required, serial CSF withdrawals were performed, and a VP Shunt was implanted according to continuous hydrocephalic dynamics with increase in ventricular width, head circumference growth crossing the percentiles and clinical symptoms accordingly, as previously described [8–11]. After discharge, all children were regularly seen at the neurosurgical outpatient clinic — usually every 3 months during the first year of life.

### Statistics

Statistical analyses were performed using SPSS (Version 25, IBM Corporation, USA), and figures were prepared using PRISM (Version 9.1.1, GraphPad Software, USA). Normal distribution was assessed by interpretation of histograms, of z-values for skewness and kurtosis and the Shapiro–Wilk normality test. Non-normally distributed values are presented as median and range (minimum to maximum).

## Results

### Patient cohort

During the 10-year study period 116 infants underwent a neuroendoscopic procedures. The underlying primary condition leading to subsequent neurosurgical treatment was intraventricular hemorrhage (IVH, n = 102), intraventricular infection (IVI, n = 7), cysts (n = 3), congenital malformations (n = 3), and tumor (n = 1). While most children were primarily surgically treated at our institution, some children were transferred for treatment of either hydrocephalus or acquired IVI after previous surgical interventions at their referring institution.

**Fig. 1:**
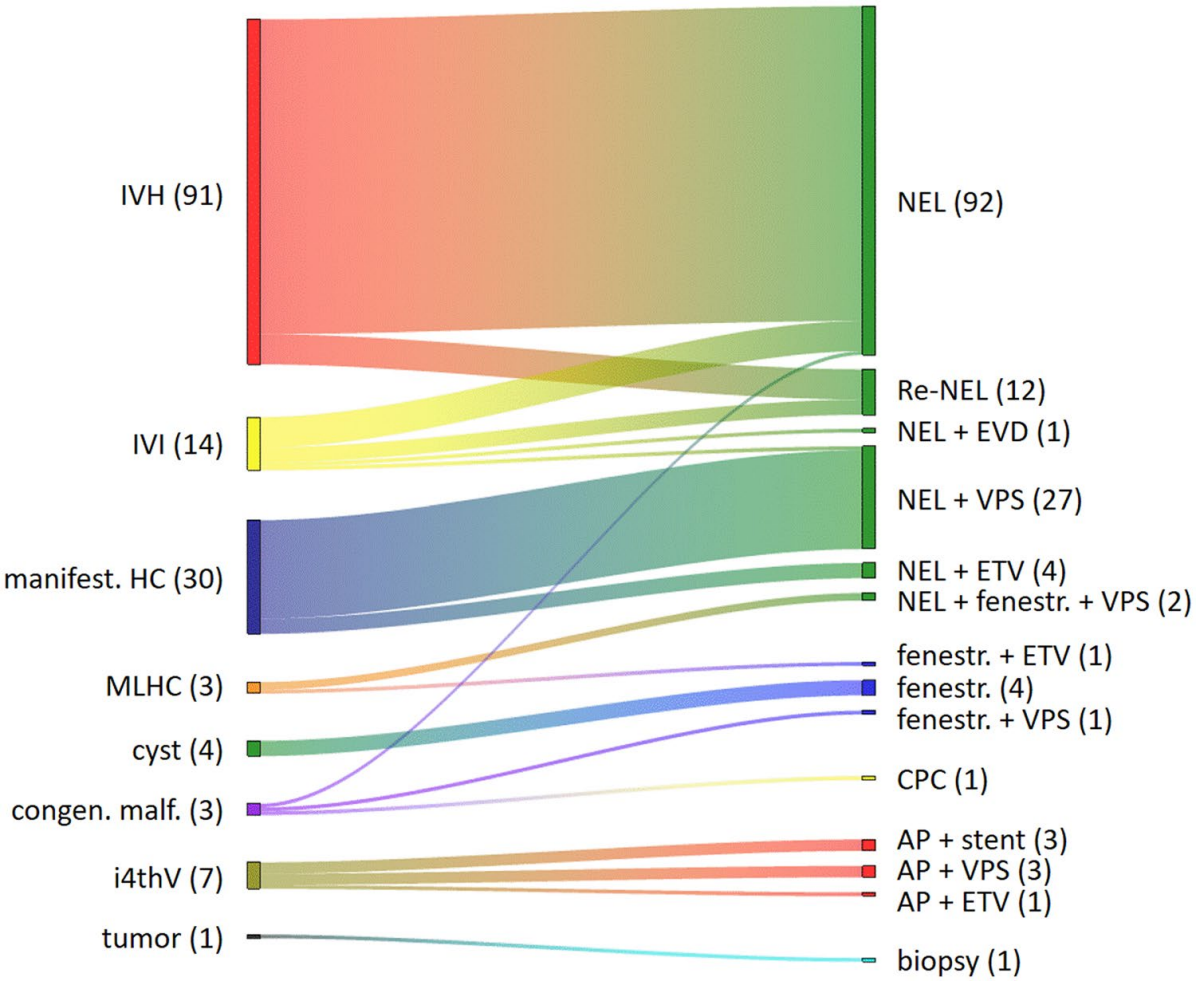
Indication for neuroendoscopic procedure (n = 153) and respective performed surgical intervention. Abbreviations: IVH, intraventricular hemorrhage; IVI, intraventricular infection; manif. HC., manifest hydrocephalus; MLHC, multiloculated hydrocephalus; malform, malformation; i4thV., isolated fourth ventricle; NEL, neuroendoscopic lavage; Re-NEL, repeated neuroendoscopic lavage; EVD, external ventricular drainage; VPS, ventricular peritoneal shunt; ETV, endoscopic third ventriculocisternostomy; fenestr., endoscopic fenestration; CPC, choroid plexus coagulation; AP, endoscopic aqueductoplasty

Among the 116 infants of the patient cohort, in total 153 neuroendoscopic procedures were performed. The most common indication at the time of the neuroendoscopic procedures was IVH (n = 91), IVI (n = 14), manifested hydrocephalus (n = 30) due to an underlying primary condition with previous other treatments (posthemorrhagic hydrocephalus (PHHC) n = 28), postinfectious hydrocephalus (PIHC, n = 1), and hydrocephalus due to congenital malformation (n = 1). Other reasons were isolated 4th ventricle (n = 7), multiloculated hydrocephalus (MLHC, n = 3), arachnoid cysts (n = 4), Dandy Walker Malformation (n = 1), Schizencephaly (n = 1), myelomeningocele with intraventricular dermoid (n = 1), and tumor (n = 1) (Fig. 1).

The total number of neurosurgical procedures (neuroendoscopic and others) during the first year of life amounted to 246. Forty-eight infants (41.4%) underwent only one surgical procedure (all neuroendoscopic), 33 infants (28.4%) underwent 2 surgical procedures, 18 infants (15.5%) underwent 3 surgical procedures, and 17 infants (14.7%) with 4 or more procedures (4 procedures (n = 11), 5 procedures (n = 3), 6 procedures (n = 2), 7 procedures (n = 1)).

**Fig. 2:**
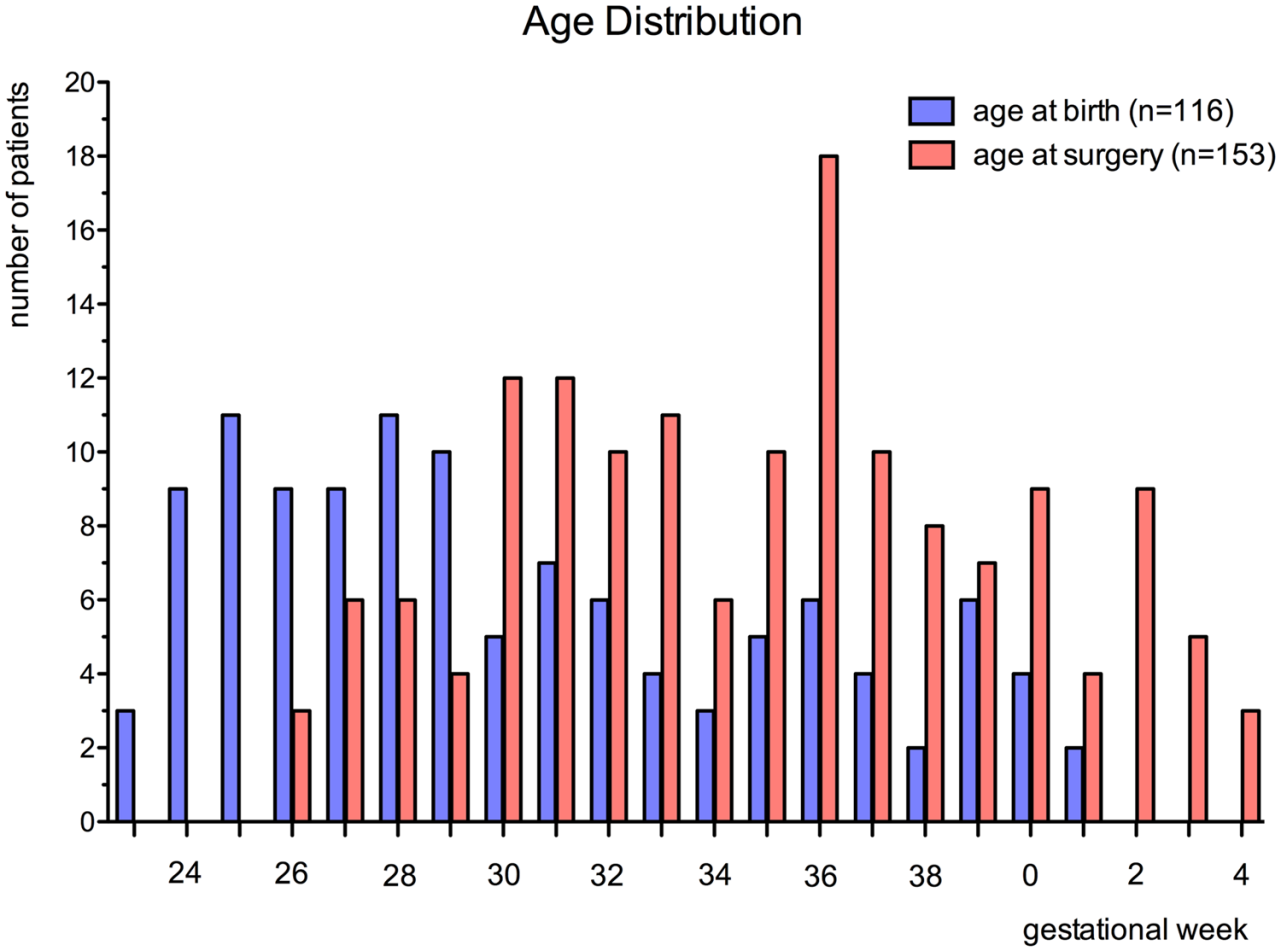
Distribution of gestational age at birth (n = 116) and postmenstrual age at the time of surgery (n = 153)

The median gestational age of the patient cohort at birth was 29 ^1^/7 weeks (range: 23 ^3^/7 weeks to 40 ^6^/7 weeks); the median postmenstrual age of the cohort at the time of the 153 neuroendoscopic procedures was 35 ^0^/7 weeks (range: 26 ^0^/7 weeks to 43 ^6^/7 weeks; Fig. 2).

### Type of surgery

Figure 1 presents the performed type of surgeries. In total 138 neuroendoscopic lavage procedures (NEL, 90%) were performed, which included clearance of the ventricular system from remnant of previous intraventricular hemorrhage or infectious debris. Of those, 92 NELs were performed as the primary procedures together with the insertion of a subcutaneous reservoir. 12 NELs were repeated lavage procedures, and 27 NELs were combined with concomitant VP shunt insertion, 2 NELs together with fenestration and VP shunt, 4 NELs are combined with an ETV, and one NEL combined with an EVD placement.

Seven neuroendoscopic procedures (5%) were performed to treat an isolated 4th ventricle by a stented aqueductoplasty, six endoscopic procedures (4%) were used to fenestrate intracranial cysts and one procedure each to coagulate the choroid plexus and to biopsy a tumor, respectively.

### Outcome

Three infants of the cohort died during the study period secondary to sequelae of extreme prematurity (2 infants with septic complications in the wake of necrotizing enterocolitis, 1 infant with severe bronchopulmonary dysplasia, and pulmonary hypertension), and one infant due to congenital malformations. These four infants underwent endoscopic lavage and one infant received a VP shunt, subsequently.

#### Intraventricular hemorrhage and infection

Within the cohort, 80 infants suffered from intraventricular hemorrhage for whom a neuroendoscopic lavage was performed as the primary surgical intervention to treat posthemorrhagic hydrocephalus. Of this subgroup 47 children required later insertion of a CSF diverting shunt (shunt rate: 58.8%). The remaining 41.2% of these children remained shunt free.

**Fig. 3:**
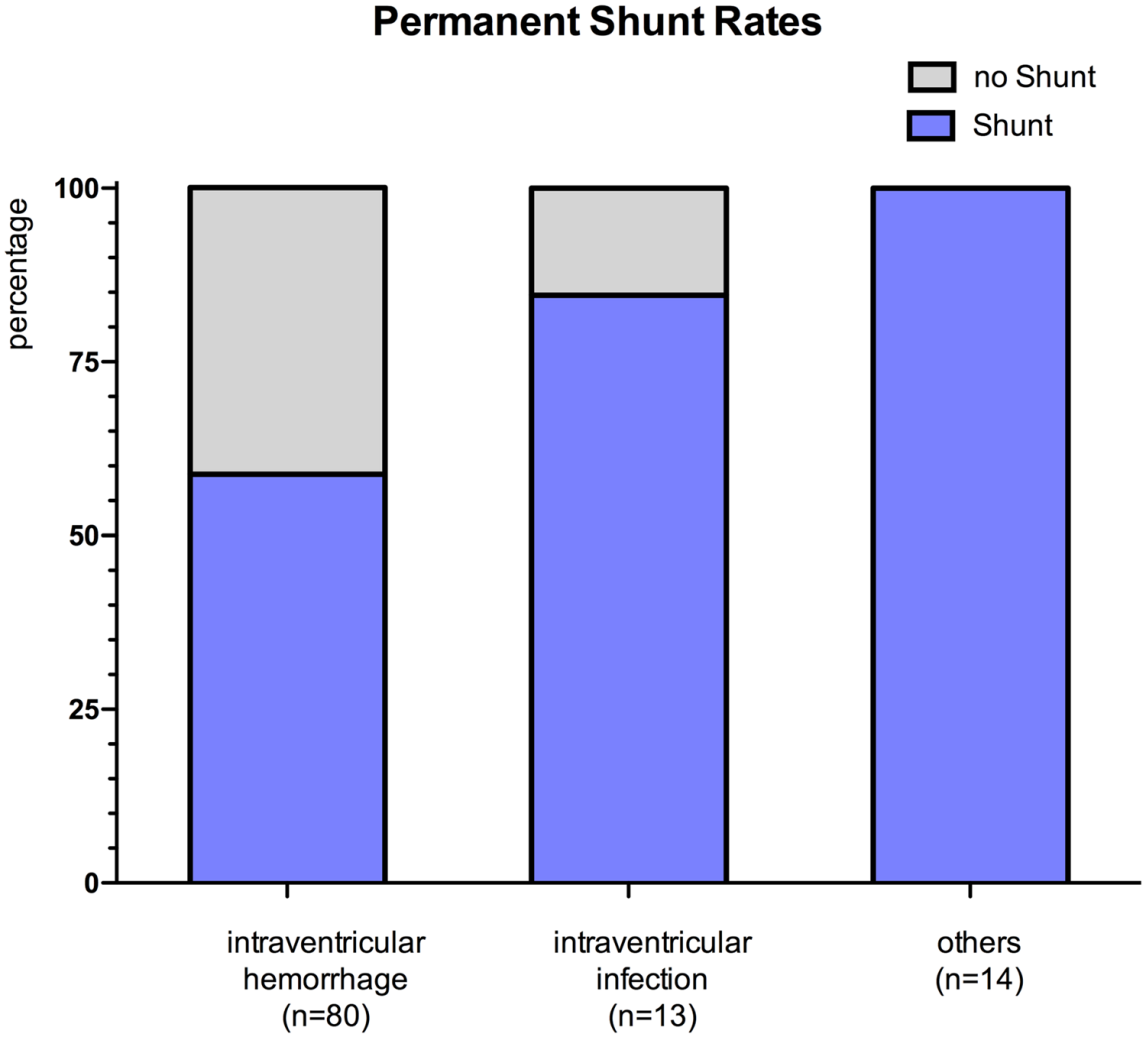
Permanent shunt rates by underlying diagnosis. (The depicted data of IVH refer to the subgroup of children who underwent NEL as the primary intervention.)

13 children had CSF infection with ventriculitis at presentation and underwent 14 NEL procedures (12 NEL with reservoir, 1 NEL with VP shunt insertion, 1 NEL with EVD). During the follow-up 11 of these children required a CSF diverting shunt (shunt rate 84.6%; Fig. 3).

#### Hydrocephalus

**Fig. 4:**
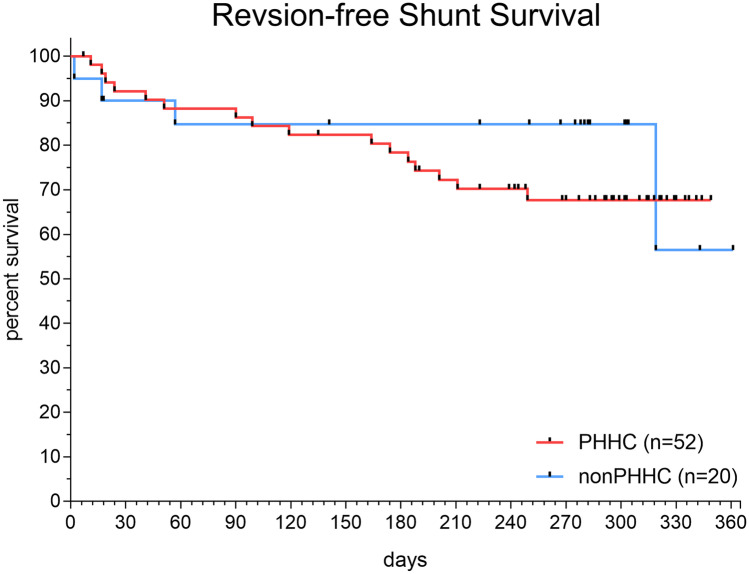
Kaplan–Meier plot showing survival of shunts (n = 72) during the first year after insertion. Patients treated by VP shunt for isolated IVH (n = 52) and for other reasons (n = 20) did not differ significantly in revision-free survival rate during 12 months of follow-up

For 6 children, an ETV was initially performed to treat hydrocephalus, no ETV was permanently successful, and all required permanent CSF diversion with a shunt within the first year of life. In one patient a pure aqueductal stenosis was treated with a stented ETV showing extensive arachnoid membranes in the prepontine cistern at time of failure. Three patients received ETV together with NEL in PHHC cases with aqueductal stenosis in which we could not see relevant blood and protein remnants during surgery. In two patients of postinfectious hydrocephalus ETV was done together with fenestrations of cystic malformations occluding the Sylvian aqueduct. The median time from ETV to shunt was 35.5 days (range: 9–195 days). In total, 72 children (62%) of the whole cohort of 116 children ultimately required permanent CSF diversion via a VP or VA shunt (VA shunt: n = 3). Twenty cases of the shunted individuals (28.8%) needed surgical shunt revision due to either shunt dysfunction (n = 11) or shunt infection (n = 9) throughout their first year of life resulting in a total of 66.7% shunt survival rate for the first year of life. The patients treated by NEL for posthemorrhagic hydrocephalus did not show inferior shunt survival (67.7%), compared to the remaining infants of the cohort treated for other underlying diseases (56.4%; Fig. 4).

#### Complications

Thirty-eight of 116 infants (32.8%) underwent 43 operative revisions for various other reasons after the 153 neuroendoscopic procedure (28.1%). Twelve secondary infections were seen in 11 infants after all procedures (4.9%) and requiring further surgery. Among those, 3 infections were seen after lavage or re-lavage while 8 infections were shunt infections and one shunt re-infection. Other experienced complications requiring surgical revision were CSF fistula (n = 9), shunt dysfunction (n = 8), failure of ETV (n = 6), subosseous dislocation of the implanted reservoir (n = 3), impaired wound healing requiring revision (n = 2), subdural hygroma (n = 2), re-enlargement of fenestrated arachnoid cyst (n = 1), and secondary intraventricular hemorrhage (n = 1).

## Discussion

The present manuscript reports on a large cohort of 116 infants who underwent a neuroendoscopic procedure prior to completion of the 4th week of life with a median age of 29 ^1^/7 weeks at birth and a median age of 35 ^0^/7 postmenstrual weeks at the time of the first surgical intervention. The presented indications for surgery and performed procedures as well as the complications establish a comprehensive overview of possibilities and difficulties of neuroendoscopic surgery in this very young age group, for which only very limited data has been published so far.

The main indications to consider a neuroendoscopic procedure in the evaluated age group of premature and neonatal children are related to intraventricular hemorrhage and subsequently developing hydrocephalus. The incidence of intraventricular hemorrhage is age dependent and its risk increases with earlier prematurity. About 29–49% of children with IVH develop disturbed CSF circulation necessitating temporary measures [12, 13]. Of those, about 29–92% require insertion of a permanent CSF diversion via a shunt [13–17]. Because of prematurity, low body weight, and contamination of CSF with solid hematoma and blood degradation products, primary CSF diversion via a VP shunt is initially not possible, and temporary CSF diversion measurements are necessary. Possible options include temporary diversion via an EVD, a ventriculo-subgaleal shunt (VSGS) or via repeated punctures of an implanted subcutaneous reservoir (ventricular access device — VAD). Based on the DRIFT study (drainage, irrigation, and fibrinolytic therapy), removal of intraventricular hematoma as a therapeutic option besides mere CSF diversion has come into attention [17, 18]. In contrast to the continuous irrigation of the ventricular system over days according to the DRIFT protocol, the concept of NEL utilizes controlled endoscopic lavage and aspiration of solid hematoma in mostly one surgical procedure. The results of this technique such as rates of shunt independency in 42% and 43.4% have been published previously and are in line with our presented findings [8, 19]. The shunt independency rate after NEL of 41.1% in this cohort seems to be promising, compared to the respective, published rates of 13.9–36.5%, 5–26% and 31.8% after VSGS, VAD alone, and EVD [11, 14, 20]. The future evaluation of an open registry for differing treatment options of posthemorrhagic hydrocephalus (TROPHY-registry) will provide further insight into the benefits and advantages of the differing treatment options and allow their comparison and identification of influencing factors, e.g., age, timing of surgery, amount of intraventricular hematoma, size of residual hematoma, subsequent shunt dependency and expected neurological outcome [21].

Two further indications for NEL were used in the present cohort such as treatment of documented infection or ventriculitis and NEL as an adjunct measure to lower CSF protein load at the time of VP shunt insertion. In an infective situation, NEL was used once the infant was medically stable and under appropriate antibiotic treatment. The primary intention of NEL in such a situation is lowering the intraventricular bacterial load as well as removal of debris documented by ultrasound or MRI. Removal of this, sometimes impressive amount of intraventricular debris might enhance the control of the infection by administered systemic antibiotic treatment and lower the risk of progressive compartmentalization of the ventricular system as well as development of multiloculated hydrocephalus (MLHC) [8, 22, 23]. Furthermore, the demonstrated effect of NEL to lower CSF protein concentration has been used prior shunt implantation [24]. The overall shunt revision-free survival rate of 66.7% and a patient rate of 28.8% from 72 infants who needed revision within the first year is acceptable for this high-risk cohort of neonates [25–27]. On contrary to the rather effective treatment of hydrocephalus by CSF diverting shunt, the failure of all performed ETVs (n = 6) to permanently control a hydrocephalic situation reflect the previously published experiences after this operative procedure in very young children [4]. In the presented cohort, ETV was applied non-consistently only in a few patients with occlusion of the major CSF pathway through the posterior fossa, which developed after either intraventricular hemorrhage, infection or a combination of both. The failure of ETV in this setting indicates that occlusion of the major CSF pathways through the posterior fossa is accompanied by a concurrent impaired CSF reabsorption capacity, while the latter might play a bigger role for hydrocephalus development.

Other indications to perform neuroendoscopy were cysts or isolated 4th ventricle. In accordance with the previously reported higher rates of necessary re-operation of fenestrated cysts in infants [28], there was one re-closure of previously fenestrated cyst wall and resulting in a re-enlargement of an intraventricular cyst and necessitating stent-secured re-fenestration. Possibly due to the primary utilization of a stent through the aqueduct to treat isolated 4th ventricle [29], neither failure was experienced nor revisions were necessary for this technique and indication.

The observed complications — e.g., CSF fistula, infection, and necessary wound revisions — reflect the specific anatomic characteristics of the neonatal patient cohort. In relation to the small head of these infants the used instruments are rather voluminous and leave a transcortical channel which possibly allows for CSF migration towards the closed wound. The usually implanted ventricular catheter further sustains this route of CSF migration. If no definite CSF diversion via an implanted shunt has been established, the subsequently increased intracranial pressure of a possibly developing hydrocephalus furthermore might challenge the surgical closure. Consequently, the risk to develop a CSF fistula or secondary impaired wound healing might be higher in this specific patient population. However, in the presented patient cohort, a few children (n = 9) developed a transcutaneous CSF fistula which was treated with re-closure and broad antibiotic coverage. Of those children, 5 infants (56%) developed a manifest CSF infection. The overall rate of documented CSF infection of 4.9% (n = 12) after a total of 246 procedures. Among those, the 3 experienced infections after NEL alone compare favorably with previously published infection rates of 15% after VSGS and 17% after VAD implantation [11]. The fact that published infection rates after VSGS and VAD are not superior to the presented cohort indicates that the addition of neuroendoscopic techniques does not necessarily increase the risk of infection.

In order to keep the complication rate low, meticulous surgical technique and comprehensive postoperative care are of paramount importance. Any attempt should be made to seal the working channel through the frontal lobe towards the ventricular system to avoid development of a subdural hygroma and direct egress of CSF towards the implanted reservoir. This can be achieved by placing a cuff of gelatine sponge around the usually implanted catheter. To avoid subosseous dislocation of the reservoir, as rarely experienced in this series, the size of the burr hole must be just as large to allow passage of the used neuroendoscopic system. Correspondingly, the the initial skin incision should be positioned to allow placement of the implant away from the subsequent suture line. Meticulous skin closure should be achieved in three layers (periosteum, subcutaneous, skin) to avoid CSF fistula. Postoperative wound care and clinical monitoring are of equal importance. Daily clinical assessments, monitoring of head circumference, and repeated cranial ultrasounds evaluate possible hydrocephalus dynamics and measures, e.g., initiation of CSF withdrawal through the implanted reservoir, should be initiated, accordingly.

### Limitations

The presented data are retrospective and a single center analysis only. Furthermore, the data are affected by an initial learning curve with increasing numbers of neuroendoscopic surgeries in this age group and gaining respective experience during the period of this study.

Although the overall number of included children is of a substantial size, age-based subgroups of the included infants would still be relatively small and subanalysis could not been performed in further detail. The included ages cover a time period of extensive development from extreme prematurity to term-born children, and it is likely that complications and outcome would be age dependent [30]. Therefore, even larger cohorts in a preferential multicenter study design are needed [21].

## Conclusion

The presented data of a relevant cohort of neonatal children indicate the neuroendoscopic techniques which are feasible in this age group. The primary indication is related to intraventricular hemorrhage and subsequent posthemorrhagic hydrocephalus and is mainly represented by neuroendoscopic lavage sometimes in combination with other surgical procedures. The reported outcomes after neuroendoscopic lavage confirm previous reports of our group with regard to shunt rate. The overall complication profile of the whole cohort after all neuroendoscopic surgeries is comparable to published neurosurgical series, indicating that neuroendoscopy appears to be safe and can be applied without increased risk for acute complications. The possible long-term benefits of these techniques in comparison to others await further evaluation in ongoing studies.

## References

[1] Bowes AL, King-Robson J, Dawes WJ, James G, Aquilina K (2017). Neuroendoscopic surgery in children: does age at intervention influence safety and efficacy? A single-center experience. J Neurosurg Pediatr.

[2] Constantini S, Sgouros S, Kulkarni A (2013) Neuroendoscopy in the youngest age group. World Neurosurg 79(2 Suppl):S23.e1–11 10.1016/j.wneu.2012.02.003 22381849

[3] Kulkarni AV, Riva-Cambrin J, Holubkov R, Browd SR, Cochrane DD, Drake JM, Limbrick DD, Rozzelle CJ, Simon TD, Tamber MS, Wellons JC, Whitehead WE, Kestle JR, Hydrocephalus Clinical Research N (2016). Endoscopic third ventriculostomy in children: prospective, multicenter results from the Hydrocephalus Clinical Research Network. J Neurosurg Pediatr.

[4] Kulkarni AV, Sgouros S, Constantini S, Investigators I (2016). International Infant Hydrocephalus Study: initial results of a prospective, multicenter comparison of endoscopic third ventriculostomy (ETV) and shunt for infant hydrocephalus. Childs Nerv Syst.

[5] Schulz M, Bührer C, Spors B, Haberl H, Thomale UW (2013). Endoscopic neurosurgery in preterm and term newborn infants–a feasibility report. Childs Nerv Syst.

[6] Cavalheiro S, Dastoli PA, Suriano IC, Sparapani F, Mello FB (2007) Brain wash in premature neonate with intraventricular hemorrhage. Paper presented at the Childs Nerv Syst

[7] Etus V, Kahilogullari G, Karabagli H, Unlu A (2018). Early endoscopic ventricular irrigation for the treatment of neonatal posthemorrhagic hydrocephalus: a feasible treatment option or not? A multicenter study. Turk Neurosurg.

[8] Schulz M, Buhrer C, Pohl-Schickinger A, Haberl H, Thomale UW (2014). Neuroendoscopic lavage for the treatment of intraventricular hemorrhage and hydrocephalus in neonates. J Neurosurg Pediatr.

[9] Kestle JR, Holubkov R, Douglas Cochrane D, Kulkarni AV, Limbrick DD, Luerssen TG, Jerry Oakes W, Riva-Cambrin J, Rozzelle C, Simon TD, Walker ML, Wellons JC, Browd SR, Drake JM, Shannon CN, Tamber MS, Whitehead WE, Hydrocephalus Clinical Research N (2016). A new Hydrocephalus Clinical Research Network protocol to reduce cerebrospinal fluid shunt infection. J Neurosurg Pediatr.

[10] Mazzola CA, Choudhri AF, Auguste KI, Limbrick DD Jr, Rogido M, Mitchell L, Flannery AM (2014) Pediatric Hydrocephalus Systematic R, Evidence-Based Guidelines Task F Pediatric hydrocephalus: systematic literature review and evidence-based guidelines. Part 2: management of posthemorrhagic hydrocephalus in premature infants. J Neurosurg Pediatr 14(Suppl 1):8–23 10.3171/2014.7.PEDS14322 25988778

[11] Wellons JC, Shannon CN, Holubkov R, Riva-Cambrin J, Kulkarni AV, Limbrick DD, W Whitehead, Browd S, Rozzelle C, Simon TD, Tamber MS, Oakes WJ, Drake J, Luerssen TG, Kestle J, Hydrocephalus Clinical Research N (2017). Shunting outcomes in posthemorrhagic hydrocephalus: results of a Hydrocephalus Clinical Research Network prospective cohort study. J Neurosurg Pediatr.

[12] Brouwer AJ, Brouwer MJ, Groenendaal F, Benders MJ, Whitelaw A, de Vries LS (2012). European perspective on the diagnosis and treatment of posthaemorrhagic ventricular dilatation. Arch Dis Child Fetal Neonatal Ed.

[13] Limbrick DD, Mathur A, Johnston JM, Munro R, Sagar J, Inder T, Park TS, Leonard JL, Smyth MD (2010). Neurosurgical treatment of progressive posthemorrhagic ventricular dilation in preterm infants: a 10-year single-institution study. J Neurosurg Pediatr.

[14] Badhiwala JH, Hong CJ, Nassiri F, Hong BY, Riva-Cambrin J, Kulkarni AV (2015). Treatment of posthemorrhagic ventricular dilation in preterm infants: a systematic review and meta-analysis of outcomes and complications. J Neurosurg Pediatr.

[15] Nagy A, Bognar L, Pataki I, Barta Z, Novak L (2013). Ventriculosubgaleal shunt in the treatment of posthemorrhagic and postinfectious hydrocephalus of premature infants. Childs Nerv Syst.

[16] Wellons JC, Shannon CN, Kulkarni AV, Simon TD, Riva-Cambrin J, Whitehead WE, Oakes WJ, Drake JM, Luerssen TG, Walker ML, Kestle JR (2009). A multicenter retrospective comparison of conversion from temporary to permanent cerebrospinal fluid diversion in very low birth weight infants with posthemorrhagic hydrocephalus. J Neurosurg Pediatr.

[17] Whitelaw A, Evans D, Carter M, Thoresen M, Wroblewska J, Mandera M, Swietlinski J, Simpson J, Hajivassiliou C, Hunt LP, Pople I (2007). Randomized clinical trial of prevention of hydrocephalus after intraventricular hemorrhage in preterm infants: brain-washing versus tapping fluid. Pediatrics.

[18] Whitelaw A, Pople I, Cherian S, Evans D, Thoresen M (2003). Phase 1 trial of prevention of hydrocephalus after intraventricular hemorrhage in newborn infants by drainage, irrigation, and fibrinolytic therapy. Pediatrics.

[19] d'Arcangues C, Schulz M, Buhrer C, Thome U, Krause M, Thomale UW (2018). Extended experience with neuroendoscopic lavage for posthemorrhagic hydrocephalus in neonates. World Neurosurg.

[20] Bock HC, Feldmann J, Ludwig HC (2018). Early surgical management and long-term surgical outcome for intraventricular hemorrhage-related posthemorrhagic hydrocephalus in shunt-treated premature infants. J Neurosurg Pediatr.

[21] Thomale UW, Cinalli G, Kulkarni AV, Al-Hakim S, Roth J, Schaumann A, Buhrer C, Cavalheiro S, Sgouros S, Constantini S, Bock HC (2019). TROPHY registry study design: a prospective, international multicenter study for the surgical treatment of posthemorrhagic hydrocephalus in neonates. Childs Nerv Syst.

[22] Deopujari CE, Padayachy L, Azmi A, Figaji A, Samantray SK (2018). Neuroendoscopy for post-infective hydrocephalus in children. Childs Nerv Syst.

[23] Schulz M, Bohner G, Knaus H, Haberl H, Thomale UW (2010). Navigated endoscopic surgery for multiloculated hydrocephalus in children. J Neurosurg Pediatr.

[24] Gaderer C, Schaumann A, Schulz M, Thomale UW (2018). Neuroendoscopic lavage for the treatment of CSF infection with hydrocephalus in children. Childs Nerv Syst.

[25] Kulkarni AV, Riva-Cambrin J, Butler J, Browd SR, Drake  JM, Holubkov R, Kestle JR, Limbrick DD, Simon TD, Tamber MS, Wellons JC, Whitehead WE, Hydrocephalus Clinical Research N (2013). Outcomes of CSF shunting in children: comparison of Hydrocephalus Clinical Research Network cohort with historical controls: clinical article. J Neurosurg Pediatr.

[26] Paulsen AH, Due-Tonnessen BJ, Lundar T, Lindegaard KF (2017). Cerebrospinal fluid (CSF) shunting and ventriculocisternostomy (ETV) in 400 pediatric patients. Shifts in understanding, diagnostics, case-mix, and surgical management during half a century. Childs Nerv Syst.

[27] Tervonen J, Leinonen V, Jaaskelainen JE, Koponen S, Huttunen TJ (2017). Rate and risk factors for shunt revision in pediatric patients with hydrocephalus-a population-based study. World Neurosurg.

[28] Schulz M, Oezkan Y, Schaumann A, Sieg M, Tietze A, Thomale UW (2021) Surgical management of intracranial arachnoid cysts in pediatric patients: radiological and clinical outcome. J Neurosurg Pediatr 1–11. 10.3171/2020.10.PEDS20839 10.3171/2020.10.PEDS20839 33930866

[29] Cinalli G, Spennato P, Savarese L, Ruggiero C, Aliberti F, Cuomo L, Cianciulli E, Maggi G (2006). Endoscopic aqueductoplasty and placement of a stent in the cerebral aqueduct in the management of isolated fourth ventricle in children. J Neurosurg.

[30] Behrens P, Tietze A, Walch E, Bittigau P, Buhrer C, Schulz M, Aigner A, Thomale UW (2020) Neurodevelopmental outcome at 2 years after neuroendoscopic lavage in neonates with posthemorrhagic hydrocephalus. J Neurosurg Pediatr 1–9 10.3171/2020.5.PEDS20211 32764179

